# Kynurenine Pathway in Diabetes Mellitus—Novel Pharmacological Target?

**DOI:** 10.3390/cells12030460

**Published:** 2023-01-31

**Authors:** Kamila Kozieł, Ewa M. Urbanska

**Affiliations:** Laboratory of Cellular and Molecular Pharmacology, Chair and Department of Clinical and Experimental Pharmacology, Medical University, 20-090 Lublin, Poland

**Keywords:** kynurenine, kynurenic acid, quinolinic acid, 3-hydroxykynurenine, indoleamine 2,3-dioxygenase, kynurenine aminotransferase, diabetes mellitus, neurodegeneration, metabolism, inflammatory

## Abstract

The tryptophan–kynurenine pathway (Trp–KYN) is the major route for tryptophan conversion in the brain and in the periphery. Kynurenines display a wide range of biological actions (which are often contrasting) such as cytotoxic/cytoprotective, oxidant/antioxidant or pro-/anti-inflammatory. The net effect depends on their local concentration, cellular environment, as well as a complex positive and negative feedback loops. The imbalance between beneficial and harmful kynurenines was implicated in the pathogenesis of various neurodegenerative disorders, psychiatric illnesses and metabolic disorders, including diabetes mellitus (DM). Despite available therapies, DM may lead to serious macro- and microvascular complications including cardio- and cerebrovascular disease, peripheral vascular disease, chronic renal disease, diabetic retinopathy, autonomic neuropathy or cognitive impairment. It is well established that low-grade inflammation, which often coincides with DM, can affect the function of KP and, conversely, that kynurenines may modulate the immune response. This review provides a detailed summary of findings concerning the status of the Trp–KYN pathway in DM based on available animal, human and microbiome studies. We highlight the importance of the molecular interplay between the deranged (functionally and qualitatively) conversion of Trp to kynurenines in the development of DM and insulin resistance. The Trp–KYN pathway emerges as a novel target in the search for preventive and therapeutic interventions in DM.

## 1. Introduction

Dietary tryptophan (Trp), apart from its structural role, is a primary source for various biologically active molecules, including serotonin, kynurenines, indoles and nicotinamide adenine dinucleotide (NAD^+^). Only minor quantities of Trp are used for the synthesis of proteins (~1%) and serotonin (2–4%), whereas the remaining 95–97% of the Trp pool is converted along the Trp–kynurenine pathway (Trp–KYN), which yields a number of intermediates termed collectively kynurenines [1,2,3,4].

Kynurenines include several biologically active metabolites displaying a wide range of biological actions that are often contrasting, such as cytotoxic/cytoprotective, oxidant/antioxidant or pro-/anti-inflammatory. The canonical classification of kynurenines presents them as either protective, such as kynurenic acid (KYNA) or toxic, such as quinolinic acid (QUIN) or 3-hydroxykynurenine (3-OH-KYN). Thus, the net biologic effect of the pathway depends on the local concentration of metabolites, cellular environment, activity of the enzymes along the path, as well as complex positive and negative feedback loops.

In the brain, the role of kynurenines has been extensively studied and is relatively well recognized. The abnormal ratio between neuroprotective and neurotoxic metabolites has been implicated in the pathogenesis of neurodegenerative disorders such as Huntington’s disease and Alzheimer’s disease, psychiatric illnesses such as schizophrenia or depression and other diseases [5]. Recent evidence suggests that the disturbed metabolism of Trp along the Trp–KYN pathway may also impact the pathogenesis of metabolic disorders, e.g., diabetes mellitus (DM) [6,7,8], cardiovascular diseases [9,10,11,12], kidney disorders [13,14] or cancer [15]. DM is a chronic, heterogeneous metabolic disease characterized by disturbed glucose metabolism and complex underlying pathophysiology. Its prevalence is steadily increasing worldwide, and, despite available therapies, successful management is often difficult to achieve. Regardless of the specific type of diabetes, microvascular, macrovascular and neuropathic complications substantially decrease life quality and result in high economic burden. Therefore, further clarification of pathogenic factors, a search for better ways to predict the development of diabetes and introduction of novel therapeutic approaches are needed. In the view of emerging data, kynurenines seem to serve as systemic integrators of energy metabolism through their impact on adipocytes, immune and muscle cells. It is well established that low-grade inflammation, which often coincides with DM, can affect the function of the Trp–KYN pathway and, conversely, that kynurenines may modulate the immune response. This review provides a detailed summary of findings concerning the status of the Trp–KYN pathway in DM based on the available animal, human and microbiome studies. We start with a description of the Trp–KYN pathway, characterize the metabolites and briefly review the features of DM. In further sections, we highlight the importance of the molecular interplay between the deranged (functionally and qualitatively) conversion of Trp to kynurenines in DM and its complications.

## 2. Tryptophan–Kynurenine Pathway

The conversion of Trp along the Trp–KYN pathway starts with the activity of step-limiting enzymes, tryptophan 2,3-dioxygenase (TDO) or indoleamine 2,3-dioxygenase (IDO), the latter of which occurs in two isoforms, IDO1 and IDO2. In the periphery, hepatic TDO regulates and determines the serum levels of Trp. IDOs impact the fate of Trp metabolism outside the liver, e.g., in the brain or the immune system. The enzymatic product, N-formyl-L-kynurenine, is rapidly converted to L-kynurenine (KYN) by kynurenine formamidase (also known as arylformamidase) [1]. Further KYN metabolism leads to the formation of diverse substances, collectively known as kynurenines, which exhibit a wide range of bioactive properties. Under physiological conditions, KYN transformations proceed preferentially along a route yielding quinolinic acid (QUIN), with the final product being nicotinamide adenine dinucleotide (NAD^+^), a coenzyme for redox reactions and therefore an important source of cellular energy. KYN is also metabolized through either the transamination by four different kynurenine aminotransferases (KATs 1–4) yielding neuroprotective KYNA or by kynureninase to anthranilic acid (AA). The QUIN branch starts with the hydroxylation of KYN by kynurenine-3-monooxygenase (KMO) generating cytotoxic 3-hydroxykynurenine (3-OH-KYN), which is subsequently hydrolyzed to 3-hydroxyanthranilic acid (3-HAA) by kynureninase. 3-HAA can also be formed as a result of AA hydroxylation. 3-OH-KYN can be also transaminated to xanthurenic acid (XA) by KATs.

**Figure 1:**
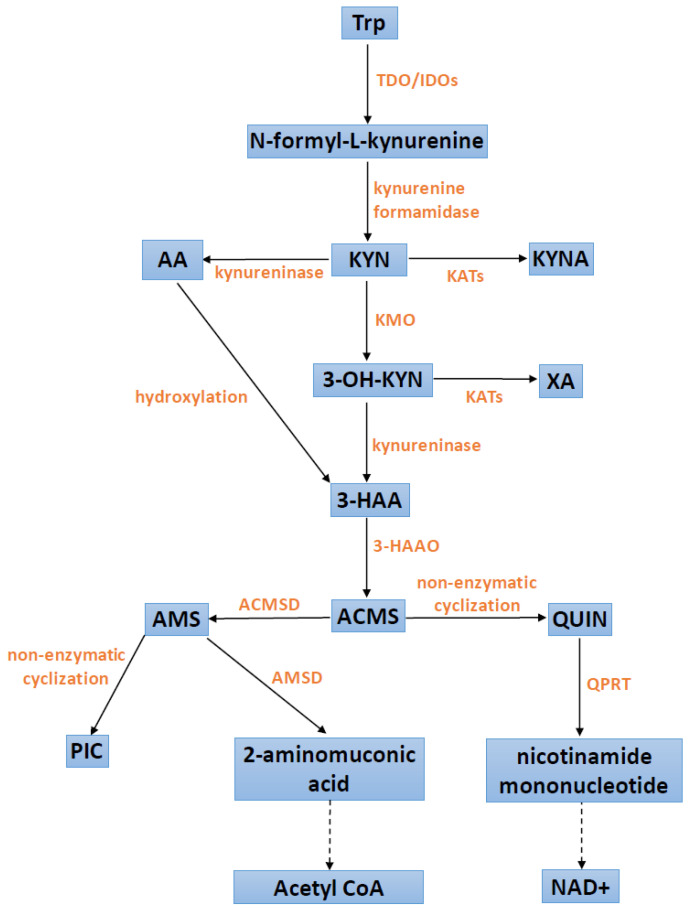
Scheme of the kynurenine pathway. 3-HAA: 3-hydroxyanthranilic acid; 3-HAAO: 3-hydroxyanthranilic acid 3,4-dioxygenase; 3-OH-KYN: 3-hydroxykynurenine; AA: anthranilic acid; acetyl CoA: acetyl coenzyme A; ACMS: 2-amino-3-carboxymuconicacid-6-semialdehyde; ACMSD: 2-amino-3-carboxymuconic acid semialdehyde decarboxylase; AMS: 2-aminomuconic-6-semialdehyde; AMSD: 2-aminomuconic semialdehyde dehydrogenase; IDOs: indoleamine 2,3-dioxygenases; KATs: kynurenine aminotransferases; KMO: kynurenine-3-monooxygenase; KYN: kynurenine; KYNA: kynurenic acid; NAD^+^: nicotinamide adenine dinucleotide; PIC: picolinic acid; QPRT: quinolonate phosphoribosyl transferase; QUIN: quinolinic acid; TDO: tryptophan 2,3-dioxygenase; Trp: tryptophan; XA: xanthurenic acid.

Further down, 3-HAA is metabolized by 3-hydroxyanthranilic acid 3,4-dioxygenase (3-HAAO) to QUIN, which is an endogenous agonist of N-methyl-D-aspartate receptors. The intermediate product, 2-amino-3-carboxymuconicacid-6-semialdehyde (ACMS) is unstable and, after non-enzymatic cyclization, spontaneously generates QUIN. In peripheral tissues and the brain, the quinolinate phosphoribosyl transferase (QPRT) converts QUIN to nicotinamide mononucleotide, whose further transformations lead to the formation of NAD^+^. As mentioned, the intermediate product of 3-HAA to QUIN transformation, 2-amino-3-carboxymuconicacid-6-semialdehyde (ACMS), is unstable and, after non-enzymatic cyclization, spontaneously generates QUIN. However, ACMS may be also further metabolized by 2-amino-3-carboxymuconic acid semialdehyde decarboxylase (ACMSD) to form 2-aminomuconic-6-semialdehyde (AMS). The latter can be spontaneously formed into neuroprotective picolinic acid (PIC) or metabolized by 2-aminomuconic semialdehyde dehydrogenase (AMSD) into 2-aminomuconic acid, depending on the extent of the substrate saturation of AMSD. Then, 2-aminomuconic acid is further degraded to acetyl CoA [2,3,16,17,18,19]. Figure 1 presents the most important steps in the Trp–KYN pathway.

The activity of rate-limiting enzymes responsible for the first step of the Trp–KYN pathway, TDO and IDOs, was linked with the regulation of immune response and, conversely, may be affected by inflammation itself [20]. Pro-inflammatory factors (e.g., IFN-γ, LPS, TNF-α) are known to induce the TDO/IDOs expression in vitro and in vivo [21,22].

Therefore, in DM, fully active or low-grade inflammation, due to enhancement of TDO/IDOs expression or activity, may shift the Trp metabolism towards the Trp–KYN pathway with an ensuing increase in the levels of Trp–KYN pathway metabolites. The final net effect will depend on the proportion of metabolites displaying disparate properties as well as a number of genetic and epigenetic factors that influence the activity of biosynthetic enzymes. Additionally, the intestinal microbiota can influence the systemic activity of the Trp–KYN pathway and thus modulate various aspects of human physiology.

## 3. Kynurenines

The intricate activities of various kynurenines impede their clear distinction into neuroprotective or neurotoxic, especially considering that their actions depend on the local availability of the substrates, enzymatic activities and broadly defined cellular milieu [22,23]. Furthermore, an intricate network of individually determined congenital factors as well as exogenous modifiers, e.g., presence of inflammation, may alter the final role played by a specific kynurenine. Brief summary of their actions is shown in Table 1.

KYN, the first stable metabolite in the Trp–KYN pathway, easily crosses the blood-brain barrier; thus, its peripheral concentration may directly affect the activity of the pathway within the brain [24]. Beneficial neuroprotective effects of KYN observed in different experimental models have been traditionally attributed to the production of KYNA. However, KYN itself exhibits redox properties. Results of recent studies showed that KYN is a scavenger of ^●^OH and ONOO^−^; thus, it can act as an endogenous antioxidant [25]. KYN was identified as an endogenous ligand of aryl hydrocarbon receptor (AhR), functioning as a target for xenobiotics and as a transcription factor [26]. AhR belongs to a family of nuclear receptors crucially involved in the regulation of gene expression and implicated, among others, in cellular differentiation and inflammation. It is widely expressed in barrier tissues, especially in immune cells, epithelial cells or endothelial cells, and it primarily contributes to immunosuppression. Activation of AhR results in reduced activity of natural killer (NK) cells, inhibition of T cell proliferation and an enhanced differentiation of naïve T cells into regulatory T cells (Tregs). Hence, KYN, through its stimulation of AhR, has been implicated as a new player in the field of immune responses that is able to reduce the degree of inflammation [27].

KYNA, a direct product of KYN transamination, is an important Trp–KYN pathway metabolite that displays pleiotropic biological activity [4]. It can be synthesized by various peripheral cells and tissues including liver, kidneys, gastrointestinal tract, endothelial and immune cells, but, unlike KYN, peripheral KYNA cannot cross the blood-brain barrier. Therefore, in the brain, KYNA is synthesized in situ, mostly within glial cells [4,28,29]. KYNA is a well-documented broad-spectrum antagonist of glutamate receptors of N-methyl-D-aspartate (NMDA), α-amino-3-hydroxy-5-methyl-4-isoxazole propionic acid (AMPA) and kainate (KA) type. KYNA displays the highest affinity for the strychnine-insensitive glycine site of NMDA receptors, with an IC_50_ ~8–15 μM in the absence of glycine and ~50–200 μM in the presence of 10 μM glycine [4]. KYNA was also reported to act as a noncompetitive antagonist at the α7 subunit of the nicotinic acetylcholine receptor (α7nAChR), although the data are not consistent [30]. At low concentrations, KYNA may also impact the presynaptic release of glutamate, dopamine or serotonin [28]. Furthermore, KYNA is an endogenous ligand of G-protein coupled orphan receptor GPR35, which is found on immune cells, in adipose tissue and in the gastrointestinal tract [31]. Upon binding to GPR35, KYNA may ameliorate inflammation through the down-regulation of TNF expression, diminished interleukin-4 (IL-4) and α-defensin secretion or inhibition of Th17 cell differentiation [32,33]. The pharmacological profile of KYNA includes, similarly to KYN, the stimulation of AhR, which results in anti-inflammatory activity [34]. In addition to various receptor-mediated effects, KYNA displays antioxidative properties, acting as a scavenger of free radicals, such as peroxynitrite (ONOO^−^), superoxide anion (O_2_^−^) and hydroxyl radicals (^●^OH) [35]. These features are beneficial in the context of metabolic diseases, which are frequently linked to the excessive generation of free radicals and oxidative stress [36]. Furthermore, recent data indicate that low doses of KYNA can exert nootropic effects, possibly through an involvement of the serotonergic, dopaminergic, α- and β-adrenergic and opiate systems [37]. These observations contrast with reports demonstrating that high KYNA levels may impair working memory and contextual learning [4] and constitute yet another argument for the beneficial role of KYNA in the brain.

Another arm of the Trp–KYN pathway leads to neurotoxic kynurenines. 3-OH-KYN, a direct product of KYN hydroxylation, is considered neurotoxic, mostly through its ability to generate free radicals and subsequent derangement of cellular proteins [3,4,38]. Upon further metabolic conversion, 3-OH-KYN yields QUIN, an agonist of the NMDA receptor that displays potent neurotoxic properties. Excessive activation of NMDA receptors by QUIN stimulates excitotoxicity and neuronal cell death in various experimental paradigms [4,38]. Furthermore, QUIN enhances reactive oxygen species (ROS) production, impairs mitochondrial function and disrupts the activity of endogenous antioxidant enzymes [39]. Emerging evidence correlates extracellular availability of QUIN with the function of immune system. Certain pro-inflammatory cytokines, such as TNFα or IL-1β, promote QUIN production, whereas anti-inflammatory IL-4 inhibits IDO/TDO, thus suppressing QUIN production [40].

Similarly, 3-HAA may induce oxidative stress and promote synthesis of ROS [22,38]. However, an anti-inflammatory and neuroprotective role of 3-HAA during inflammation has also been suggested. In astrocytes, 3-HAA can induce the expression of hemeoxygenase-1, which exhibits anti-inflammatory and cytoprotective properties [41].

XA possesses antioxidant properties and acts as a scavenger of free radicals [22]. The least-known metabolite of the Trp–KYN pathway with neuroprotective properties is PIC. It acts as an efficient chelator for metals (such as chromium, zinc, manganese, copper and iron) in the brain, preventing protein aggregation and oxidative stress [42,43]. Immunomodulatory properties of PIC a have also been shown. PIC can activate macrophage effector functions and induce macrophage inflammatory proteins (MIPs) [44].

**Table 1:** The major effects of selected Trp–KYN pathway metabolites. 3-HAA: 3-hydroxyanthranilic acid; KYN: kynurenine; KYNA: kynurenic acid; QUIN: quinolinic acid; PIC: picolinic acid; XA: xanthurenic acid.

Metabolite	Bioactive Properties	Reference
KYN	antioxidant, immunomodulating	[25,26]
KYNA	neuroprotective, immunomodulating, antioxidant	[2,4,30,45]
3-OH-KYN	neurotoxic, generates free radicals	[2,4]
3-HAA	neurotoxic, generates free radicals, possibly also neuroprotective and anti-inflammatory through induction of hemeoxygenase-1	[2,41]
QUIN	excitotoxic, generates free radicals	[19,39]
PIC	neuroprotective, chelator	[22]
XA	antioxidant, scavenger of free radicals	[22]

## 4. Tryptophan–Kynurenine Pathway in Human Diseases

The net effect of kynurenines on living organisms depends on their local concentration, cellular environment and complex positive and negative feedback loops. The activation of the Trp–KYN pathway is suggested to be a physiological reaction to stressors such as inflammation, infection, metabolic disturbances, aging and others [23]. Activation of the pathway usually results in the disproportional stimulation of metabolic routes. Thus, the imbalance between beneficial and harmful kynurenines has been implicated in the pathogenesis of various disorders. Alteration in the levels of kynurenines and the activities of their biosynthetic and metabolizing enzymes was observed in a wide range of illnesses, including neurological, psychiatric, neoplastic and autoimmune disorders [45,46,47,48].

In various neuropsychiatric disorders, including mood disorders and schizophrenia [49], excessive activation of the Trp–KYN pathway may lead to reduced synthesis of serotonin, a neurotransmitter essential for the modulation of mood and cognition [50,51]. Accumulating data reveal abnormalities in kynurenines among patients with major depression, anxiety disorder or schizophrenia [52,53,54,55]. However, the data on peripheral and central changes in metabolites are still unclear [4]. In neurodegenerative diseases, including Huntington’s and Parkinson’s disease, the majority of findings indicate an abundance of neurotoxic kynurenines, especially QUIN and 3-hydroxykynurenine, and a deficiency of KYNA within specific areas of brain. The decline in KYNA and malfunction of the neuroprotective arm of the pathway may generate virtually identical consequences as an excessive production of QUIN and other neurotoxic kynurenines. In line with these observations, switching from the neurotoxic branch of the KP that yields QUIN, to the neuroprotective branch that produces KYNA was suggested to bring beneficial effects [4]. Altered Trp–KYN metabolism seems to result, at least in part, from genetic changes, as, for example, it was suggested that the development of Parkinson’s disease may be influenced by certain single nucleotide polymorphisms (SNPs) of IDO1 [56]. The data concerning Alzheimer’s disease are not conclusive and there are discrepancies between results from animal models, human cerebrospinal fluid and brain tissue samples. In general, however, lower levels of neuroprotective KYNA are the most consistent finding in neurodegenerative diseases and psychiatric disorders [57,58].

Apart from the direct impact of certain kynurenines on cellular survival, their biological effects comprise indirect modulation of cell viability through the immune system. The essential role of the Trp–KYN pathway in suppression of immune processes is broadly documented, with KYN highlighted as one of the most potent immunosuppressive kynurenines [46]. Under immune stimulation, the upregulation of TDO/IDOs is a mechanism which initiates a vicious cycle. Produced kynurenines may induce immune tolerance which can be detrimental for the course of sepsis, certain viral infections, obesity, aortic aneurysm formation or tumor development [20,46]. On the other hand, reduction of an excessive immune response may be beneficial during autoimmune conditions [16]. Immunosuppressive effects of kynurenines are exhibited mainly by the functional modulation of immune cells, including T cells and natural killer (NK) cells. Kynurenines promote anti-inflammatory responses by shifting the Th1/Th2 cell ratio towards Th2. Enhanced IDO1 expression or administration of KYN may reduce the proliferation and induce apoptosis of antigen-specific T cells, stimulate the generation of T regulatory cells (Tregs) or suppress the function and proliferation of NK cells [40].

Chronic inflammation is a common finding linked to DM and has been implicated as an important factor underlying unfavorable course of the disease and its various complications [24,27,28]. Low-grade inflammation may affect the function of the Trp–KYN pathway, and it was suggested to influence the ontogenesis of diabetes [59]. The following sections of this review present the basics of DM pathology and the Trp–KYN pathway alterations observed in experimental animal models, human studies and microbiome research.

## 5. Diabetes Mellitus

Diabetes mellitus (DM) is a chronic metabolic disease characterized by elevated glucose levels. In 2021, the global DM prevalence in the population between 20 and 79 years of age was estimated as 10.5% (536.6 million people), and it is predicted to rise up to 12.2% (783.2 million) in 2045 [60]. According to the American Diabetes Association (ADA), diabetes is classified into the following categories: DM type 1 (DMT1), DM type 2 (DMT2), gestational DM and other specific types of DM with various pathologies (e.g., monogenic diabetic syndromes, the exocrine pancreas diseases and drug-/chemical-induced diabetes) [60]. Serious macro- and microvascular complications including coronary heart disease, stroke and peripheral vascular disease, end-stage renal disease, retinopathy, neuropathy or cognitive impairment can result from DM [61].

### 5.1. Diabetes Mellitus Type 1

DMT1 results from an autoimmune destruction of β-cells, which ultimately causes an absolute insulin deficiency and subsequent hyperglycemia [62]. DMT1 may be diagnosed at any age, yet it occurs mostly in childhood and adolescence [63]. Maternal obesity is associated with a higher risk of DMT1 in the offspring [64].

The etiology of DMT1 is still not completely understood. According to the classical hypothesis for DMT1 development, one or multiple environmental triggers activate the immune system among individuals with a genetic predisposition [65]. Produced autoantibodies, directed against specific β-cell proteins, initiate cellular destruction. The majority of patients diagnosed with DMT1 have at least one of the following antibodies present: against insulin, against 65 kDa glutamic acid decarboxylase (GAD65), against insulinoma-associated protein 2 (IA-2) or against zinc transporter 8 (ZnT8). However, most people with only one of these antibodies do not develop DMT1 [63,66]. Nonetheless, recent studies suggest that β-cells themselves may contribute to DMT1. Individuals at risk of DMT1 may have abnormal β-cells, which become a target for the immune system. According to this “β-cells centric hypothesis”, an inflammatory state developing in the pancreas leads to higher expression of human leukocyte antigen (HLA) class I molecules in β-cells, making them detectable by cytotoxic CD8^+^ T cells. In a healthy organism, regulatory T cells (Tregs) are responsible for the repression of these autoreactive T cells. However, patients with DMT1 exhibit a reduced suppressive capacity [65]. Additionally, patients with cancer treated with immune checkpoint inhibitors seem to be at higher risk of DMT1 development due to an enhanced immune response and decreased immunosuppression [67].

Recent reports show an increased incidence of new-onset diabetes probably linked to SARS-CoV-2 infection. COVID-19 might increase the risk of developing diabetes, potentially by causing β-cell injury, an excessive inflammatory response, activation of the renin-angiotensin-aldosterone system or lifestyle changes during the pandemic [68]. On the other hand, diabetes itself increases the risk of severe/fatal SARS-CoV-2 infection, probably due to an impaired immune response [69].

DMT1 is commonly diagnosed in youth, a time when the central nervous system undergoes rapid developmental alterations. The young brain is especially susceptible to the profound changes of blood glucose levels. The episodes of hypoglycemia caused by the therapy may be responsible for the brain dysfunction found in diabetic patients. Multiple studies have shown that individuals with DMT1 frequently display deficits in cognitive domains, including general intelligence, mental flexibility, memory, attention, information-processing speed, psychomotor efficiency, and visual perception. Brain structure can also be affected by diabetes. DMT1 patients have reduced gray and white matter volumes. Diminished gray matter volume was associated with poor glycemic control and high frequency of severe hypoglycemic events [70,71].

### 5.2. Diabetes Mellitus Type 2

DMT2 is the most common type of diabetes (more than 95% of all cases), and it is caused by insulin resistance and defective insulin secretion due to pancreatic β-cell dysfunction [72,73]. In contrast to DMT1, DMT2 is relatively rare among young people. However, there were approximately 41,600 new cases of DMT2 in children and adolescents in 2021 worldwide, suggesting that DMT2 is no longer “adult-onset diabetes” [73]. In studies carried out on DMT2 children with co-existing obesity, insulin resistance and β-cell dysfunction were similarly prevalent as in older patients [74].

DMT2 is a multifactorial disease with risk factors including: genetic predisposition, environmental influences, age, obesity, sedentary lifestyle, hypertension and high triglycerides levels, smoking and gestational diabetes [75]. Additionally, childhood severe acute malnutrition has been associated with metabolic changes in adulthood, including greater risk of DMT2 [76].

Insulin resistance is defined as a decreased sensitivity and responsiveness to insulin. It impairs the ability of physiologically insulin-sensitive cells to take up glucose and triglycerides, which results in high glucose and triglyceride blood levels [77]. Insulin resistance can develop in multiple organs, especially in skeletal muscle, heart, liver and adipose tissue [78]. It is a strong risk factor not only for DMT2 but also for the development of cardiovascular diseases. The occurrence of insulin resistance is determined mainly by unhealthy lifestyles and obesity, but genetic factors may also contribute to its development [79].

Dysfunction of β-cells in DMT2 mostly follows hyperglycemia and hyperlipidemia, which are both associated with an excessive nutrition state, as is often observed in obesity. Under these conditions, β-cells are exposed to deleterious factors, such as inflammation, endoplasmic reticulum dysfunction, metabolic stress or amyloidosis, which altogether may lead to a loss of islet integrity [72]. Deteriorating β-cells undergo rapid apoptosis, leading to a decrease in their overall mass with proinsulin and amyloid fibrils as specific markers of the cells’ destruction. Furthermore, amyloid fibrils may accumulate in the diabetic pancreas, thus potentiating β-cell apoptosis [78]. Insulin resistance and β-cell dysfunction synergistically enhance the degree of hyperglycemia and culminate in a relative deficiency of insulin [80].

Recent evidence implies the role of the gut microbiota in the pathogenesis of DMT2. *Ruminococcus*, *Fusobacterium* and *Blautia* were shown to be positively associated with DMT2, while *Bifidobacterium*, *Bacteroides*, *Faecalibacterium*, *Akkermansia* and *Roseburia* were negatively associated with DMT2 [81]. Although *Lactobacillus* is frequently reported in studies in DMT2 patients, the results are discordant [82]. It seems that *Lactobacillus*’ effects on DMT2 may be species-specific or strain-specific [83,84,85]. Gut microbes may affect DMT2 in a few ways: by modulation of inflammation [86,87], by influencing gut permeability [88,89], by influencing glucose metabolism and IR [90] and by influencing fatty acid oxidation and energy expenditure [91]. The gut microbiota can also contribute to the effectiveness of drug therapy in DMT2. Pharmacotherapy can modulate the microbiota and improve DM. On the other hand, microbes can affect pharmacokinetics and pharmacodynamics of the drugs [92].

Patients with DMT2, similarly to DMT1, can experience drug-therapy-related severe hypoglycemia, which impairs cognition and was linked with the prevalence of dementia [71]. Cognitive dysfunction in DMT2 mainly includes deficits in the domains of memory, psychomotor speed, and executive function. The risk of dementia is increased in elderly diabetic patients. DM-related brain pathology may increase risk of Alzheimer’s disease. People diagnosed with DMT2 have lower gray and white matter volumes. Some studies have suggested that atrophy may be augmented in the hippocampus [70]. The hypothalamus also has been reported to be affected by DM [93].

### 5.3. Gestational Diabetes Mellitus

Gestational diabetes mellitus (GDM) is defined as diabetes diagnosed in the second or third trimester of pregnancy [60]. According to International Diabetes Federation estimates, the global prevalence of GDM in 2021 was 14.0% [94]. Risk factors of GDM development include mother’s obesity, unhealthy diet, advanced age, genetic factors, and a family history of IR or diabetes. The majority of GDM cases (about 80%) is similar to DMT2, presenting as IR and β-cell dysfunction. The consequences of GDM apply to both mother and child. GDM is associated with antenatal or postnatal depression, and it also increases the risk of preterm birth and preeclampsia. Additionally, in many GDM patients a Caesarean section is required. Women with GDM are at high risk for development of DMT2 in the future. The GDM short-term consequences for the fetus include fetal overgrowth, excessive insulin production, which contributes to β-cell dysfunction and IR, and increased risk of hypoglycemia after the delivery. Studies show that GDM also increases the risk of stillbirth. In the long term, children born to women with GDM are at higher risk of obesity, DMT2, cardiovascular disease, and other metabolic diseases [95,96].

## 6. The Tryptophan–Kynurenine Pathway and Diabetes Mellitus in Animal Studies

### 6.1. Periphery

Analyses of peripheral levels of the Trp–KYN pathway metabolites as well as measurements of the respective enzymes have been performed in various experimental settings. Available data support the notion that kynurenines are involved in the development of DM [97]. Alterations in kynurenines have been detected in animal models of DM, and the direct effects of kynurenines on the glucose metabolism have been shown in vitro and in vivo. Furthermore, modulation of carbohydrate metabolism by the manipulations of the Trp–KYN pathway enzymes was demonstrated (Table 2).

Classical ways to induce experimental DM comprise high-fat diet as well as genetically or chemically evoked dysfunction of the pancreas. Administration of streptozocin (STZ), leading to a progressive destruction of pancreatic β-islets, or a combination of low-dose STZ and high-fat diet provide commonly used models of DMT2 [98]. Other models include the spontaneously diabetic Torii (SDT) rats, Zucker diabetic fatty (ZDF) rats or the Otsuka Long-Evans Tokushima Fatty (OLETF) rats [99].

Initial studies of diabetogenic effects of some kynurenines were observed already in the 1960s and were performed on naïve animals. XA was found to form complexes with insulin and to reduce its activity, thus predisposing rats to diabetes [100]. Furthermore, it may inhibit insulin secretion form the pancreas and induce pancreatic β-cell apoptosis through a caspase-3-dependent mechanism [27]. In contrast, a conversion of the XA precursor, 3-OH-KYN, to 3-HAA by kynureninase may lower the risk of DM. However, this process is disrupted by pyridoxal-5-phosphate (vitamin B6) deficiency [101].

The majority of available data suggest that KYNA exerts antidiabetic properties. KYNA, through stimulation of GPR35 receptors and increased AMPK phosphorylation, reduces inflammation and insulin resistance, as shown in adipocytes and muscle cells [28]. Furthermore, this mechanism was implicated in controlling the regulation of energy expenditure exerted by KYNA [28]. Others, however, demonstrated that KYNA and XA may inhibit proinsulin synthesis in isolated rat pancreatic islets [102]. Furthermore, KYNA, through antagonism of NMDA receptors in the dorsal vagal nucleus [103] could enhance hepatic glucose secretion.

On the other hand, plasma Trp and KYNA are reduced already prior to the development of DM, as shown in SDT and OLETF rats [99]. In the model of rat GDM, a lower level of Trp and KYNA was detected in both maternal serum and amniotic fluid samples. In contrast, the urinary KYNA level was increased 1.8-fold in diabetic monkeys compared to non-diabetic ones [104]. Untargeted metabolomic studies concerning other kynurenines revealed that peripheral levels of KYN and AA were reduced in serum from diabetic dogs [105].

Chronic inflammation is now considered one of the key factors underlying the pathogenesis of diabetes, cardiovascular disease or psychotic and mood disorders [106]. The reduction of NAD biosynthesis, possibly as a result of impaired conversion of 3-HAA to nicotinic acid, was observed in diabetic rabbits [107,108]. Reduced activity of hepatic TDO, but unchanged intestinal IDO, as well hepatic and kidney KMO were reported in the model of DM induced by alloxan in rabbits [109]. Trp metabolism initiated by IDO in dendritic cells (DCs) emerged as major mechanism of peripheral tolerance [110]. DCs lacking functional IDO can acquire a tolerogenic phenotype via paracrine production of kynurenines by IDO-competent DCs [110]. Non-obese diabetic (NOD) mice, susceptible to the development of autoimmune diabetes modeling DMT1, manifest defective IDO expression in DCs [111]. Furthermore, IDO-expressing fibroblasts were able to inhibit the progression of diabetes in NOD mice when injected intraperitoneally in the amount of 15 × 10^6^ (15M). Such treatment also resulted in higher plasma KYN levels compared to mice treated with a lower dose of IDO-expressing fibroblasts (10M) cells, in which reversion of hyperglycemia was unsuccessful [112]. These data imply that enhanced IDO1 expression in DCs can restore immunoregulatory signaling in autoimmune diabetes.

Furthermore, overexpression of IDO1 in TGF-β-treated plasmacytoid DCs (pDCs) from NOD mice resulted in these cells’ ability to suppress the in vivo presentation of a pancreatic β-cell autoantigen [113]. IDO-mediated immunosuppression also affects Tregs. In vitro, Tregs induced by Trp starvation and kynurenines protected non-obese severe combined immunodeficiency (NOD-SCID) mice from an induction of DM [114]. Similarly, Tregs generated in a microenvironment with low Trp and a mixture of kynurenines could protect mice from the disease in a model of fulminant DM [110]. Enhanced IDO activity may also reduce the viability of macrophages and their pro-inflammatory activity, probably by the reduction in the available Trp pool and inhibition of inducible nitric oxide synthase (iNOS) expression [115]. Beneficial effects of IDO were confirmed under in vitro conditions in immature human dendritic cells (iDC) inoculated with a chimeric fusion protein vaccine containing the pancreatic β-cell auto-antigen proinsulin linked to a mucosal adjuvant, the cholera toxin B subunit (CTB-INS). Such a vaccine induced prominent upregulation of IDO1, bringing about the inhibition of DC maturation and subsequent immunological tolerance [116].

The mechanism underlying IDO-mediated immunosuppression most probably involves enhanced generation of KYN. Thus, inhibition of KYN breakdown along the Trp–KYN pathway should also exert beneficial effects in DM. In such a scenario, a deficit of KYN could contribute to the development of autoimmune DM. However, the data on actual levels of KYN in experimental models of DM are rather scarce. In high-fat diet and STZ-induced DMT2, a significant decreases in the urine concentrations Trp and KYN were observed [98]. A pharmacological approach aimed at boosting KYN levels and based on KMO knockdown or pharmacological inhibition revealed that the reduction of KMO activity enhanced glucose-stimulated insulin release and improved glucose metabolism under in vitro and in vivo conditions, respectively [117]. KYN was shown to potentiate glucose-induced insulin secretion by normal islets [118]. However, an increased conversion of the Trp load to KYN and QUIN was reported in hepatocytes obtained from STZ-induced diabetic rats [119,120,121]. Possibly, an increased response of the Trp–KYN pathway to pro-inflammatory stimuli in DM could be viewed as a protective measure aimed at restoring proper carbohydrate metabolism and not as the element of pathogenesis.

Interestingly, the efficacy of KYN as a novel suppressive adjuvant for a DMT1 vaccine was evaluated. Co-immunization of KYN and the GAD65 phage vaccine resulted in the prevention of hyperglycemia in 60% of NOD mice for at least one month compared to the GAD65 phage vaccine alone [62]. It was demonstrated that KYN promoted Foxp3^+^ Treg induction, suppressed dendritic cell maturation and GAD65-specific T cell proliferation, significantly increased IL-10, IL-4 and TGF-β1 and decreased IFN-γ and IL-2 in the NOD mouse model [62]. Thus, apart from selective stimulation of IDO-1, KMO inhibition could become a potential therapeutic strategy for DMT2.

Relatively little is known about the role of other enzymes along the Trp–KYN pathway in DM pathophysiology. Peripheral KATs activity was studied in some DM models. Renal, but not hepatic, KATs and kynureninase activities were reduced in diabetic-hyperlipidemic rabbits [109]. Arylformamidase (kynurenine formamidase), coded for by the *Afmid* gene, hydrolyzes N-formyl-L-kynurenine to form KYN [122,123]. *Afmid*-knockout mice exhibit impaired glucose tolerance with unchanged insulin sensitivity, suggesting involvement of arylformamidase in glucose-induced insulin secretion. Interestingly, higher KYNA and XA were detected in *Afmid*-knockout mice urine [124].

Kynureninase shifts the metabolism of 3-OH-KYN to 3-HAA at the cost of potentially diabetogenic XA formation. Zebularine is a cytidine analog and a methyl transferase inhibitor used as a pharmacological tool to induce the expression of kynureninase. The compound evokes a long-lasting suppression of immune destruction of pancreatic islet allotransplants in STZ-induced diabetic rats [125]. Possibly, the observed cytoprotection resulted from the reduced synthesis of XA [125]. However, this conclusion is not supported by other data. The hepatocytes nuclear factor 1α (Hnf1α) gene is associated with the development of Maturity-onset Diabetes of the Young type 3 (MODY3). Studies in *Hnf1α*-null mice demonstrated an actual decrease of urinary XA levels without any significant changes in urinary Trp, KYN or KYNA levels [126,127].

The results from animal research on peripheral levels of kynurenines seem to implicate the protective role of KYN in DM. Most data also suggest an antidiabetic role of KYNA, but further detailed research, including prospective analysis of KYN and KYNA status prior and during DM, is necessary.

### 6.2. Brain

Data on the central changes in the Trp–KYN pathway in the course of experimental diabetes-mimicking scenarios are rather limited (Table 2). Hyperglycemia, a key symptom of diabetes, does not influence KYNA synthesis in rat brain cortical slices. However, hyperglycemia may significantly enhance the inhibitory effects of mitochondrial toxins and D,L-homocysteine on KYNA production in the rat brain in vitro. These results suggest that under hyperglycemic conditions, mitochondrial dysfunction and high concentrations of D,L-homocysteine may hamper the synthesis of neuroprotective KYNA in the brain, thus leading to central complications of diabetes [126]. In vivo, the hippocampal, but not cortical or striatal, KYNA concentration was increased in rats with STZ-induced DM either untreated or treated with insulin (220% and 170% of CTR, respectively). The activity of KAT I was not affected by DM brain structures, whereas KAT II activity was increased in the cortex and hippocampus but not in the striatum of diabetic animals. Insulin treatment normalized cortical but not hippocampal KAT II activity [127]. An increase in IDO expression has also been observed in the hippocampus of diabetic rats [128]. Similarly, in STZ-induced DM in mice, increased IDO expression in hypothalamic astrocytes was detected [129].

The potential impact of increased brain KYNA levels could be associated with two aspects of diabetic pathology. It may either reflect the activation of endogenous mechanisms aimed at neuroprotection, or it may have a negative impact on cognition. Furthermore, hyperglycemia is partly due to an increased hepatic glucose production, which, in rodents, is partially controlled by the hypothalamus. The activation of NMDA receptors in the dorsal vagal complex (DVC) was shown to lower hepatic glucose production in rats [103]. It could be assumed that a selective increase in KYNA in some brain regions could lead to an increase in hepatic glucose synthesis via inhibition of NMDA receptors in the DVC, but more research is needed on this subject.

**Table 2:** Changes in enzymes and metabolites of Trp–KYN pathway in diabetes mellitus. 3-HAA: 3-hydroxyanthranilic acid; AA: anthranilic acid; DCs: dendritic cells; DM: diabetes mellitus; DMT1: diabetes mellitus type 1; GAD65: glutamic acid decarboxylase 65; GDM: gestational diabetes mellitus; IDO: indoleamine 2,3-dioxygenase; IFN-γ: interferon gamma; KATs: kynurenine aminotransferases; KMO: kynurenine-3-monooxygenase; KYN: kynurenine; KYNA: kynurenic acid; MODY3: maturity-onset diabetes of the young type 3; NOD: non-obese diabetic mice; OLETF: Otsuka Long-Evans Tokushima Fatty rats; pDCs: plasmacytoid dendritic cells; QUIN: quinolinic acid; SDT: spontaneously diabetic Torii rats; STZ: streptozocin; TDO: tryptophan 2,3-dioxygenase; TGF-β: transforming growth factor beta; Tregs: T regulatory cells; Trp: tryptophan; XA: xanthurenic acid.

Type of the Animal Study	Studied Model/Main Characteristics of the Study	Findings Regarding the Trp–KYN Pathway		References
		Enzymes	Metabolites	
In vitro	Isolated rat pancreatic islets	IDO1 expression is not constitutive but is activated by pro-inflammatory cytokines	KYN/KYNA production ratio is enhanced following exposure to IFN-γ; KYN potentiates the glucose-induced insulin secretion by normal islets	[102]
			KYNA and XA may inhibit proinsulin synthesis	[118]
	Mouse dendritic cells (DCs)	DCs lacking functional IDO can acquire a tolerogenic phenotype via paracrine production of kynurenines by IDO-competent DCs		[110]
	TGF-β-treated plasmacytoid DCs (pDCs) from NOD mice	pDCs acquire the ability to suppress the in vivo presentation of a pancreatic β-cell autoantigen		[113]
	Tregs induced by Trp starvation and kynurenines	These Tregs protect mice from diabetes in vivo		[110,114]
	KMO knockdown or pharmacological inhibition	Enhancement of the glucose-stimulated insulin release and improvement of glucose metabolism ^1^		[117]
Periphery	Experimental diabetes by XA in rats		XA reduces insulin activity	[100]
	STZ-induced DM in rats		↑ Liver KYN ↑ Liver QUIN	[119]
	Alloxan-induced DM in rabbits		Impaired conversion of 3-HAA to nicotinic acid	[107]
	Alloxan-induced DM in rabbits fed with high-fat diet	↓ Liver TDO ↓ Renal KATs activity ↓ Kynureninase activity in kidneys		[109]
	SDT rats and OLETF rats		↓ Plasma Trp and KYNA ^2^	[99]
	Diabetic monkeys		↑ Urinary KYNA	[104]
	NOD mice		Nicotinamide prevents T1DM in prediabetic NOD mice and reverse the pathology in hyperglycemic mice	[106]
		Defective IDO expression in DCs		[111]
		IDO-expressing fibroblasts can inhibit the progression of diabetes		[112]
	DMT1 vaccine		Co-immunization of KYN and GAD65 phage vaccine resulted in the prevention of hyperglycemia in 60% of NOD mice for at least one month	[62]
	Diabetic dogs		↓ KYN ↓ AA	[108]
	*Afmid*-knockout mice		KYNA and XA detected in urine	[124]
	Rat model of GDM		↓ TRP and KYNA in maternal serum and amniotic fluid samples	[130]
	*Hnf1α*-null mice (mimic MODY3)		↓ Urinary XA	[131]
Brain	Hyperglycemia		No direct influence on KYNA synthesis in rat brain cortical slices; enhancement of the inhibitory effects of mitochondrial toxins and D,L-homocysteine on KYNA production in rat brain in vitro	[126]
	STZ-induced DM in rats	↑ KAT II activity in cortex and hippocampus	↑ Hippocampal KYNA	[127]
		↑ Hippocampal IDO expression		[128]
	STZ-induced DM in mice	↑ IDO expression in hypothalamic astrocytes		[129]

^1^ Studied in both in vitro and in vivo conditions. ^2^ Observed already prior do the development of DM. ↓—decrease, ↑—increase.

## 7. The Tryptophan–Kynurenine Pathway and Diabetes Mellitus in Human Studies

### 7.1. Diabetes Mellitus Type 1

Under in vitro conditions, in healthy human pancreatic islets exposed to inflammatory cytokines (e.g., IFN-γ and TNF-α), the accumulation of KYN and KYNA in cells and media is strongly increased, possibly due to IDO upregulation [132,133].

Results of in vivo studies mostly indicate KYN deficiency in patients with DMT1. Higher excretion of KYN in urine was demonstrated in a group of 56 children with DMT1 [6]. However, the serum levels of kynurenines were not evaluated [6]. Others reported lower serum Trp and KYN levels in 34 DMT1 patients treated with insulin but not in DMT2 patients [134]. Similarly, in a cohort of 165 pediatric patients with DMT1, defective Trp catabolism was associated with a specific IDO1 genotype and weaker production of KYN in response to IFN-γ [135,136]. In a case-control study with 175 mother/child T1D cases (median age 5.8, range 0.7–13.0 years) and 552 controls, the KYN/Trp ratio in umbilical cord blood was shown to be associated with pre-pregnancy obesity [64].

In a group of 15 patients with DMT1 but not in 30 DMT2 patients, compared with 24 control individuals, plasma AA levels were elevated, which was possibly due to riboflavin deficiency [135,137]. Riboflavin may also alter homocysteine synthesis and thus affect the production of KYNA in the brain [138,139] or influence the synthesis of active pyridoxal-5-phosphate, a cofactor for various enzymes of Trp–KYN pathway [140].

### 7.2. Diabetes Mellitus Type 2

In general, DMT2 patients often show enhanced Trp metabolism with reduced Trp and elevated levels of downstream metabolites along the Trp–KYN pathway [7]. Overproduction of kynurenines may be induced by chronic stress or chronic low-grade inflammation, as is often observed in DMT2 individuals [7]. The results, however, are ambiguous. Mostly, a positive correlation of Trp, KYN and KYNA with disease susceptibility is shown. In a metabolomics study including 5181 participants from the cross-sectional Metabolic Syndrome in Men study, the levels of KYNA and XA were shown to correlate with a decrease both in insulin secretion and insulin sensitivity as well as with an increased susceptibility to DMT2 [141]. Analysis of associations between circulating levels of eleven Trp metabolites and the incidence of DMT2 was studied in 9180 participants of diverse racial/ethnic backgrounds from five cohorts. Trp, KYN, KYNA, XA and QUIN were positively associated with DMT2 risk, and multiple host genetic variants, dietary factors, gut bacteria and their potential interplay associated with these DMT2-related metabolites were identified [141,142]. In a randomly selected sub-cohort of 641 patients and 251 incident cases diagnosed during 3.8 years of median follow-up, baseline Trp, KYNA, KYN, QUIN and 3-HAA were associated with changes in homeostatic model assessment for insulin resistance (HOMA-IR) from baseline to one year [143]. A study in 2519 individuals with coronary artery disease (CAD) without DM observed for a median of 7.6 years, during which 173 cases of new DMT2 were identified, revealed that the urine, but not plasma, KYN/Trp ratio was strongly associated with DMT2 risk [144]. In two cohorts comprising 856 individuals with DMT2, the serum KYN/Trp ratio was associated with mortality among patients [145]. A small study including 20 males with DMT2 and 20 healthy individuals showed decreased Trp and increased 3-OH-KYN, KYNA, 3-HAA and XA in diabetic patients [146]. In a study in a group of 128 obese women including 65 normoglycemic and 63 DMT2 subjects, significantly higher Trp, KYNA, KYNA/QUIN ratio and KYNA/3-OH-KYN ratio values were detected among diabetic patients [147]. Plasma concentrations of KYN, KYNA and XA, but not Trp, were increased in a group of 30 DMT2 patients taking metformin compared to non-diabetic subjects [7].

Thus, most available data indicate that patients with DMT2 have an increased KYN/Trp ratio, which may reflect increased IDO activity, especially among individuals with poor glycemic control. Diabetic patients with good glycemic control seem to have a similar KYN/Trp ratio to healthy individuals [148,149]. However, an inverse association between IDO and DMT2 was also reported [150]. Furthermore, no difference either in the levels of kynurenines or in the expression of Trp–KYN pathway enzymes between diabetic and non-diabetic obese women enrolled in Biological Atlas of Severe Obesity from the D.E.S.I.R. cohort (a case-cohort study with 212 diabetic and 836 randomly sampled individuals) and from the ABOS cohort (n  =  100) was observed [151]. However, KYN levels were positively associated with HOMA2-IR and HOMA2-B at inclusion and with fasting insulinemia both at inclusion and at evaluation after nine years. Moreover, the expression of IDO1, kynureninase, KMO and KAT III [CCBL2] was increased in the omental adipose tissue of women with obesity compared to lean individuals, and their expression was induced by pro-inflammatory cytokines in human primary adipocytes [151].

Since lifestyle influences the risk of developing DMT2 and other metabolic diseases, studies focused on physical activity and its impact on the human organism have been carried out. A small study including 10 young males demonstrated that one hour of endurance exercise or one hour of strenuous high-volume resistance exercise increased circulating KYNA levels [152]. In contrast, sprint interval exercise elicited an early increase in KYN and a late rise in the KYNA level in 10 elderly healthy subjects (mean age 64 years) but not in 10 healthy young adults (mean age 24 years) [153]. A study in skeletal muscle and plasma from men with normal glucose tolerance (N = 12) or DMT2 (N = 12) performed at rest after acute exercise and during recovery revealed that basal mRNA expression of KAT1 and KAT2 was modestly reduced in DMT2 patients. In response to exercise, mRNA expression of KAT4 decreased. Exercise was shown to increase plasma KYNA and to reduce KYN in control individuals and DMT2 participants. Plasma Trp was reduced, and the KYNA/KYN ratio increased in both groups at recovery [154]. Since activation of GPR35 can improve energy metabolism and inflammation in mice fed a high-fat diet [155], an increased KYNA through activation of GPR35 in adipose tissue may increase energy expenditure, thus ameliorating insulin resistance.

**Figure 2:**
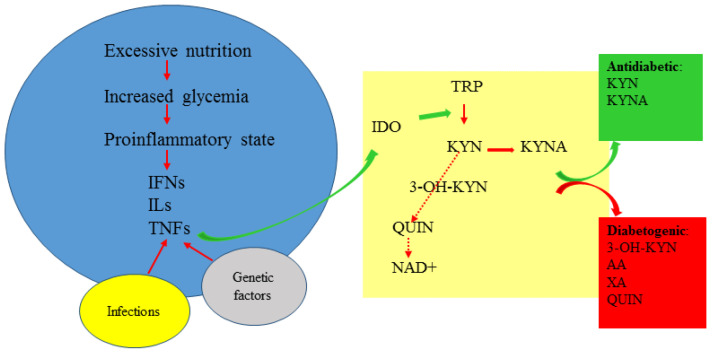
The kynurenine pathway in diabetes. 3-OH-KYN: 3-hydroxykynurenine; AA: anthranilic acid; IDO: indoleamine 2,3-dioxygenase; IFNs: interferons; ILs: interleukins; KYN: kynurenine; KYNA: kynurenic acid; NAD^+^: nicotinamide adenine dinucleotide; QUIN: quinolinic acid; TNFs: tumor necrosis factors; Trp: tryptophan; XA: xanthurenic acid.

The observational nature of clinical studies analyzing the existence of correlations between kynurenines and clinical parameters in pre-diabetes or diabetes undoubtedly indicates the existence of disturbed Trp metabolism, but, unfortunately, it does not clarify whether the changes are causative or secondary to the disease. However, when analyzed together with experimental research, accumulating data seem to indicate that in diabetes, the proportion between potentially beneficial KYNA and diabetogenic kynurenines is of importance and depends on genetic factors, physical activity, metabolic status, the degree of pancreas destruction and insulin resistance (Figure 2 and Table 3).

### 7.3. Gestational Diabetes Mellitus

GDM is a predisposing condition or prediabetes, which manifests during pregnancy [156]. One of the main risk factors for GDM is maternal obesity. 3-OH-KYN and KYN concentrations in urine samples collected in the first trimester from obese women were significantly lower in individuals who developed GDM (N = 29) than in normoglycemic women (N = 25), while Trp levels were higher [157]. Thus, these data contrast with the majority of results from studies in DMT1 and T2 patients (Table 3).

**Table 3:** Changes in enzymes and metabolites of the tryptophan–kynurenine pathway observed in human diabetes studies. 3-HAA: 3-hydroxyanthranilic acid; 3-OH-KYN: 3-hydroxykynurenine; AA: anthranilic acid; DMT1: diabetes mellitus type 1; DMT2: diabetes mellitus type 2; GDM: gestational diabetes mellitus; HLA: human leukocyte antigen; HOMA-IR: homeostatic model assessment for insulin resistance; IDO: indoleamine 2,3-dioxygenase; KATs: kynurenine aminotransferases; KYN: kynurenine; KYNA: kynurenic acid; QUIN: quinolinic acid; Trp: tryptophan; XA: xanthurenic acid.

Type of Diabetes	Characteristics of the Study	Enzymes	Metabolites	References
DMT1	Human pancreatic cells exposed to inflammatory cytokines in vitro		↑ KYN and KYNA	[132,133]
	Pediatric patients	Defective Trp catabolism associated with a specific IDO1 genotype	↑ Urinary KYN	[6,136]
	Patients treated with insulin		↓ Trp and KYN	[134]
	Mother/child cases		KYN/Trp ratio in umbilical cord blood was associated with the DMT1 high-risk HLA genotype (heterozygous DQ2/DQ8) but not with DMT1 prevalence in children	[64]
	Patients with DMT1 compared to DMT2		↑ Plasma AA	[135]
DMT2	5181 participants from the cross-sectional Metabolic Syndrome in Men		Levels of KYNA and XA correlated with a decreased insulin secretion and insulin sensitivity and with increased susceptibility to DMT2	[141]
	Participants of diverse racial/ethnic backgrounds from five cohorts		Positive correlation of Trp, KYN, KYNA, XA and QUIN with DMT2 risk	[142]
	Sub-cohort of patients from the PREDIMED Trial		Baseline Trp, KYNA, KYN, QUIN and 3-HAA were associated with changes in homeostatic model assessment for insulin resistance (HOMA-IR) from baseline to one year	[143]
	2519 patients with coronary artery disease		Urinary KYN/Trp ratio was strongly associated with DMT2 risk	[144]
	856 patients with DMT2		Serum KYN/Trp ratio was associated with mortality	
	Small study including 20 males with DMT2		↓ Trp ↑ 3-OH-KYN, KYNA, 3-HAA and XA	[146]
	Obese women		↑ Trp, KYNA, KYNA/QUIN ratio and KYNA/3-OH-KYN ratio	[147]
	Patients taking metformin		↑ KYN, KYNA and XA	[7]
	A mendelian randomization study	IDO1 was inversely associated with diabetes		[150]
	Obese women enrolled in Biological Atlas of Severe Obesity	No difference between diabetics and non-diabetics	No difference between diabetics and non-diabetics	[151]
	Acute exercise	↓ KAT1 and KAT2 expression at rest; ↓ KAT4 expression in response to exercise	↑ Plasma KYNA and ↓ KYN after exercise ^1^; ↓ plasma Trp and ↑ KYNA/KYN ratio at recovery ^1^	[154]
GDM	Obese women in the first trimester of pregnancy		↑ Urinary Trp ↓ Urinary 3-OH-KYN and KYN	[157]

^1^ Changes observed in both diabetic and control groups. ↓—decrease, ↑—increase.

## 8. Diabetes Mellitus and Microbiome Studies

The gut microbiota consists of plenty of microorganisms, including bacteria, fungi and archaea. It is essential to human health due to its involvement in various physiological activities including host metabolism, control of energy homeostasis, immune system regulation, digestion and vitamin synthesis [158]. Disturbed function of the human microbiome is implicated in multiple disorders, including obesity and diabetes [88,159,160].

Trp can be metabolized in the gastrointestinal tract through three pathways: direct metabolism by microorganisms, the Trp–KYN pathway and the serotonin pathway. The Trp–KYN pathway in the gut is functional in both immune and epithelial cells. The composition of the gut microbiota influences Trp metabolism, as the microbes can regulate the Trp–KYN pathway by their impact on IDO activity. Additionally, several bacteria have been found to encode enzymes homologous to the Trp–KYN pathway enzymes present in mammalian cells [38,161].

A number of studies concerning the status of microbiota in DM were performed (Table 4). In a cross-sectional study including 50 healthy control individuals and 161 individuals with DMT1, categorized into three groups based on the degree of the albuminuria, the fecal microbiota composition and 31 plasma metabolites were analyzed [162]. Patients with DMT1 were characterized by abnormal gut microbiota accompanied by higher plasma levels of KYN and lower plasma Trp in the group of individuals with macroalbuminuria compared to those with normoalbuminuria [162]. Correspondingly, a study in 23 individuals with HIV infection, 16 with DMT2, 21 with both conditions and 24 controls revealed that the combination of HIV and DMT2 was linked to reduced gut microbiota diversity and an increased plasma KYN/Trp ratio [163]. Another study in 28 women with HIV infection and 20 HIV-uninfected women, of which 16 HIV-positive and 6 HIV-negative individuals had also DMT2, did not show any differences in bacterial diversity associated with diabetic status. However, four genera (*Finegoldia*, *Anaerococcus*, *Sneathia* and *Adlercreutzia*) were less abundant in diabetic women compared to non-diabetics. Similarly to previous studies, in diabetic individuals, the plasma KYN/Trp ratio was higher [149].

Pregnancy also involves compositional changes of the maternal gut microbiome. These gut microbiome changes seem to shift IDO-dependent Trp metabolism toward KYN production, intestinal inflammation and gestational IR. In the study on gestational IR in mice, a significant variation in gut microbiome alpha diversity was observed throughout pregnancy. Metabolomics revealed increased plasma KYN levels at G15/19 in three different strains of pregnant mice, and intestinal IDO1 expression was increased at G15, which was associated with mild systemic and gut inflammation [164]. Pharmacologic and genetic inhibition of IDO1 lowered the levels of KYN and reversed pregnancy-associated insulin resistance. Fecal microbial transplants revealed that IDO1 induction and local KYN level effects result from the gut microbiome [164]. Significant changes in gut microbiome profiles were also observed in a GDM rat model. The ratio of *Firmicutes* to *Bacteroidetes* decreased. *Lactobacillus* and *Bacteroides* were negatively correlated with serotonin level and positively correlated with KYN level, whereas *Clostridium XlVa* and *Ruminococcus* were positively correlated with serotonin level. The KYN/Trp ratio increased significantly in the serum and prefrontal cortex, implying a switch of Trp metabolism from serotonin to the KYN pathway. The expression of IDO was upregulated in the colon and brain [165].

Since gut microbiome changes may be involved in the pathophysiology of diabetes, administration of probiotics and prebiotics has been studied in animal models and in clinical scenarios. The presence of the intestinal commensal bacteria *Lactobacillus johnsonii* was shown to inversely correlate with diabetes development in BioBreeding diabetes-prone (BBDP) rats [166]. Feeding *Lactobacillus johnsonii* N6.2 to BBDP rats decreased the incidence of diabetes development and was followed by changes in the native gut microbiome, mucosal proteins, oxidative stress response and a decrease in IFN-γ and TNF-α levels [167]. This treatment also lowered intestinal IDO gene transcription and decreased plasma KYN levels in BBDP rats. In vitro studies showed that *L. johnsonii* N6.2 produces H_2_O_2_, which strongly inhibits IDO activity [168].

A study in 61 pediatric DMT1 patients receiving *Lactobacillus rhamnosus* GG for three months revealed a significant increase in serum Trp levels in comparison with 25 control patients treated with placebo. Probiotic administration increased serum Trp levels but did not affect the levels of KYN, 3-OH-KYN, KYNA or QUIN [169]. A double-blind, randomized clinical trial in 42 healthy individuals with no known risk factors for DMT1 showed that administration of *L. johnsonii* N6.2 led to an increase of serum Trp levels and to a reduction in the KYN/Trp ratio. Moreover, an increase of circulating effector Th1 cells (CD45RO^+^CD183^+^CD196^−^) and cytotoxic CD8^+^ T cell subsets was observed in the *L. johnsonii* N6.2 group [170].

**Table 4:** Microbiome research in the context of diabetes mellitus and the tryptophan–kynurenine pathway. 3-OH-KYN: 3-hydroxykynurenine; BBDP: BioBreeding diabetes-prone rats; DMT1: diabetes mellitus type 1; DMT2: diabetes mellitus type 2; GDM: gestational diabetes mellitus; HIV: human immunodeficiency virus; IDO: indoleamine 2,3-dioxygenase; IR: insulin resistance; KYN: kynurenine; KYNA: kynurenic acid; QUIN: quinolinic acid; Trp: tryptophan.

Type of Diabetes	Characteristics of the Study	Microbiome	Metabolites and Enzymes of the Trp–KYN Pathway	References
DMT1	Patients with different degree of the albuminuria	Abnormal gut microbiota	↓ Plasma Trp ^1^ ↑ Plasma KYN ^1^	[162]
	Pediatric patients receiving *Lactobacillus rhamnosus* GG for three months		↑ Serum Trp unchanged levels of KYN, 3-OH-KYN, KYNA and QUIN	[169]
	Healthy individuals with no known risk factors for DMT1 receiving *Lactobacillus johnsonii* N6.2		↑ Serum Trp ↓ KYN/Trp ratio	[170]
	BioBreeding diabetes-prone (BBDP) rats	The presence of the intestinal commensal bacteria *Lactobacillus johnsonii* was inversely correlated with diabetes development;		[166]
		feeding *Lactobacillus johnsonii* N6.2 to BBDP rats decreased the incidence of diabetes development	↓ Intestinal IDO ↓ Plasma KYN	[167]
DMT2	Patients with HIV infection	Reduced gut microbiota diversity;	↑ Plasma KYN/Trp ratio	[163]
		no differences in gut microbiota diversity associated with diabetic status; *Finegoldia*, *Anaerococcus*, *Sneathia* and *Adlercreutzia* were less abundant in diabetic women compared to non-diabetics regardless of infection status	↑ Plasma KYN/Trp ratio	[149]
GDM	Gestational IR in mice	A significant variation in gut microbiome alpha diversity was observed throughout pregnancy	↑ Plasma KYN at G15/19; ↑ intestinal IDO1 expression associated with mild inflammation	[164]
	Rat model of GDM	↓ *Firmicutes*/*Bacteroidetes* ratio; *Lactobacillus* and *Bacteroides* positively correlated with KYN level	↑ KYN/Trp ratio in serum and prefrontal cortex; ↑ IDO expression in colon and brain	[165]

^1^ In the group of individuals with macroalbuminuria compared to those with normoalbuminuria. ↓—decrease, ↑—increase.

## 9. The Kynurenine Pathway in Disorders Related to Diabetes Mellitus

### 9.1. Obesity as a Risk Factor of DMT2

Obesity (body-mass index [BMI] ≥ 30 kg/m^2^), the strongest risk factor for DMT2, is linked with chronic low-grade inflammation and increases the probability of insulin resistance [171].

Correlations between the Trp–KYN pathway and obesity have been frequently observed. Metabolism of Trp is altered in the adipose tissue of obese people, with a significant increase in KYN production [97]. Obesity is associated with an increase in intestinal IDO activity. Interestingly, genetic deficiency of IDO or its inhibition improves insulin sensitivity and regulates lipid metabolism in liver and adipose tissue in mice. IDO1-knockout mice receiving a high-fat diet had less white adipose tissue and lower plasma leptin than wild type animals on the same diet [172]. BMI has been shown to positively correlate with KYN, KYNA and QUIN levels in plasma. In 2383 participants from the Framingham Offspring cohort, BMI was associated with 69 of 217 metabolites, including KYN and KYNA [151,173,174]. Metabolomic analysis of 188 metabolites in a group of 59 non-diabetic women (lean, overweight and obese) showed associations of altered plasma levels of some amino acids and KYN with adiposity markers. The KYN/Trp ratio has also been positively associated with BMI, fat mass, adipose tissue area and subcutaneous adipocyte size [175]. KYN level was also associated with waist circumference [176].

Bariatric surgery not only causes large weight loss but also improves or completely eliminates diabetes in previously obese patients diagnosed with DMT2. Interestingly, bariatric surgery leads to downregulation of the Trp–KYN pathway. IDO activity and levels of all metabolites of the Trp–KYN pathway, except for AA, decrease during postoperative weight loss (3 months or 12 months after the surgery) [177,178]. Another study showed that only Trp, KYNA and XA were significantly decreased in 3 months after bariatric surgery in DMT2 patients [179].

### 9.2. Complications of Diabetes Mellitus—Animal and Human Studies

#### 9.2.1. Diabetic Retinopathy

It has been proposed that the Trp–KYN pathway plays an important role in the inflammatory damage of the diabetic retina. IFN-γ and IDO levels have been shown to be higher in retinas of patients with diabetic retinopathy compared to non-diabetic retinas. It has been demonstrated that the absence of IDO inhibits capillary degeneration in diabetic mice [180].

There are two forms of diabetic retinopathy: non-proliferative diabetic retinopathy and proliferative diabetic retinopathy. IDO expression seems to be more increased in PDR patients than in NPDR subjects. Serum levels of KYN, KYNA and 3-OH-KYN have been shown to be higher in patients with diabetic retinopathy than healthy controls and higher in proliferative diabetic retinopathy patients compared to the non-proliferative form of disease. Proliferative diabetic retinopathy patients had higher serum levels of KYN, KYNA and 3-OH-KYN when compared to non-proliferative retinopathy [181]. KYN was identified as a potential marker of diabetic retinopathy progression in DMT2 patients [182].

#### 9.2.2. Diabetic Cataract

In a study with diabetic rats, lenticular levels of IFN-γ mRNA, IDO mRNA, IDO activity, Trp and KYNA increased significantly at the time of cataract onset and remained elevated through 60 days. These data suggest that locally produced IFN-γ induced production of IDO and consequently activated the Trp–KYN pathway [183].

Elevated levels of KYNA were also found in senile nuclear human cataracts [184]. Analysis of the fluid from the anterior chamber of the eye has shown that diabetic patients with cataracts have significantly higher concentrations of KYN and KYNA compared to those with cataracts alone, while concentrations of Trp and other metabolites ratios did not differ between the studied groups [185].

#### 9.2.3. Diabetic Kidney Disease (Diabetic Nephropathy)

KMO seems to be associated with albuminuria, as observed in *Kmo* gene knockout zebrafish [186]. In diabetic mice and patients diagnosed with diabetic nephropathy, reduced KMO expression in glomeruli was also observed [186].

Patients diagnosed with chronic kidney disease displayed a number of changes in the function of the Trp–KYN pathway [186]. Circulating Trp level was found to be inversely correlated with stages of diabetic nephropathy, whereas IDO activity and the levels of KYN, KYNA and QUIN were shown to increase with the progression of the disease [187,188]. Plasma KYN and KYNA have been identified as promising markers for assessment of renal functions [189,190]. Administration of renin-angiotensin-aldosterone system inhibitors resulted in a decline of serum KYN levels in patients with diabetic nephropathy compared to the untreated group [191]. Moreover, the KYN/Trp ratio was suggested as a predictive factor of angiotensin receptor blocker responsiveness in patients with diabetic kidney disease [192]. Interestingly, chronic administration of losartan (angiotensin receptor blocker) normalized hippocampal KYNA content in diabetic rats treated concomitantly with insulin [193].

#### 9.2.4. Diabetic Ketoacidosis

Diabetic ketoacidosis is a serious complication of untreated DMT1 characterized by high production of serum ketones in response to insulin deficiency and insulin resistance. Ketone bodies formed during non-treated diabetes may exert neuroprotective effects. Indeed, a ketone body, β-hydroxybutyrate (BHB) significantly enhanced KYNA production in rat brain cortical slices under conditions resembling acute diabetic ketosis in vitro. Experiments in glial cells showed that BHB stimulates the expression of KATs, which could be responsible for the enhanced KYNA synthesis [194].

A clinical study in a group of 15 children and adolescents with diabetic ketoacidosis assessed various kynurenines at 6–12 h after initiation of therapy, two weeks, and three months following ketoacidosis treatment [8]. The results demonstrated that Trp, KYN, QUIN and PIC were lower in early treatment and subsequently increased [8].

#### 9.2.5. Cognitive Impairment and Dementia

In mice with DM induced by STZ combined with a high-fat diet, the levels of KYN in both the hippocampus and serum were increased, whereas both brain and serum levels of KYNA were decreased. Concentrations of 3-OH-KYN and QUIN in the hippocampus were also higher. The activities of KATs, key enzymes for KYNA formation, were significantly decreased in skeletal muscle [61]. Others, however, showed that in the hippocampi of diabetic rats, the KYNA level was increased in both untreated groups and groups treated with insulin [127]. In the model of cortical infarct induced by permanent middle cerebral artery occlusion in male diabetic and non-diabetic mice, brain and serum QUIN levels and the QUIN/KYNA ratio were significantly increased at six weeks after infarct compared to controls. The highest levels of neurotoxic QUIN were detected in stroke-diabetic mice. Additionally, the authors performed a retrospective human study involving 23 stroke patients, 13 of them showing post-stroke cognitive impairment. The initial serum KYN and QUIN levels, QUIN/KYNA ratio and IDO activity were significantly higher in patients who presented a cognitive dysfunction [195].

In 24 patients at the early (up to 24 h after infarct) stage of stroke, serum KYNA and homocysteine levels were similar to controls, yet the KYNA level correlated positively with the level of homocysteine. Interestingly, high homocysteine is suggested as a risk factor for cognitive decline [196]. However, the role of homocysteine in the pathogenesis of DM-related complications is not clearly defined, despite intense research in this field [197]. In DMT2 patients, associations between levels of KYN, KYNA, XA and 3-HAA and lower odds of cognitive impairment were observed. No such associations occurred in individuals with normal glucose metabolism. These findings could suggest a protective role for selected kynurenines against DM-related cognitive dysfunction [198].

#### 9.2.6. Impaired Wound Healing

It has been suggested that KYNA may play a role the inflammation and fibroproliferation during the wound healing process. The targeted metabolic profiling of wounds in diabetic vs. non-diabetic mice showed that diabetes alters the metabolic profile of both uninjured skin and wounds. Uninjured diabetic skin contained twofold more KYNA than the uninjured non-diabetic skin. KYNA was one of the metabolites that displayed a significant response to injury in non-diabetic wounds but not in diabetic ones [199].

#### 9.2.7. Neuropathic Pain

Neuropathic pain is a common complication of diabetes. The KYN/Trp ratio is increased in DMT1 patients with neuropathic pain compared to diabetic controls, and it positively correlates with pain intensity. Patients with neuropathic pain also have higher KAT activity [200].

## 10. Future Perspectives and Conclusions

Accumulating evidence from animal and human research reveals an intricate network of correlations between Trp–KYN pathway products and the metabolism of carbohydrates. The results from animal research are not fully clear, possibly due to certain limitations of studied models that only partially mimic the whole spectrum of changes preceding and associated with development of diabetes in humans. However, the role of KYNA and KYN as potential anti-diabetic compounds is rather well substantiated.

Clinical studies mostly indicate KYN deficiency in patients with DMT1. In contrast, DMT2 patients often show enhanced Trp metabolism with reduced Trp and elevated levels of downstream metabolites along the Trp–KYN pathway. These observations may reflect increased IDO activity, especially among individuals with poor glycemic control. Considering the different etiologies of DMT1 and DMT2, an interesting picture arises. Acute deficiency of immunomodulatory KYN and KYNA seems to constitute one important pathological factor in the development of autoimmune DMT1, possibly due to the defective mechanisms controlling immune surveillance. In contrast, in the course of longer-lasting development of DMT2, which is accompanied by chronic low-grade inflammation, the conversion of Trp to kynurenines is enhanced. Indeed, the analyses of the Trp/KYN ratio seem to be a promising approach in predicting the occurrence of DMT2 [144], with increased KYN levels indicating higher probability of the disease. Interestingly, the beneficial effect of physical exercise seems to involve increased plasma KYNA and reduced KYN in DMT2 patients [154].

Consequently, a number of diagnostic and therapeutic options require detailed investigation. First of all, the longitudinal assessments of Trp and Trp–KYN pathway metabolites in the urine and serum of patients predisposed to DM and analyses of the genetic profile, including studies of the activity of enzymes catalyzing conversion of Trp along the Trp–KYN pathways in different populations, should be performed in order to clarify the issue of potential time-based associations between kynurenines and the development of DM.

Secondly, the Trp–KYN pathway has emerged as a potential therapeutic target in DM. In fact, administration of KYNA was shown to reduce the hyperlipidemia-evoked inflammation and insulin resistance in skeletal muscle and adipocytes [201], and encouraging results were obtained with KYN used as a novel suppressive adjuvant for a DMT1 vaccine [62]. Therefore, the molecular interplay between Trp catabolism along the Trp–KYN pathway and the development and progression of DM and insulin resistance emerges as a novel target in the search for preventive and therapeutic interventions in DM. However, considering the broad range of functions covered by kynurenines, the precise interventions aimed to reduce pancreatic destruction, to limit insulin resistance and to impact the complications of DM will require the use of precise and selective pharmacological tools. This challenging but exciting approach may change the future prophylaxis and therapy of DM.
